# COL2A1 Is a Novel Biomarker of Melanoma Tumor Repopulating Cells

**DOI:** 10.3390/biomedicines8090360

**Published:** 2020-09-18

**Authors:** Bhavana Talluri, Kshitij Amar, Michael Saul, Tasnim Shireen, Vjollca Konjufca, Jian Ma, Taekjip Ha, Farhan Chowdhury

**Affiliations:** 1 Biomedical Engineering Program, School of Electrical, Computer, and Biomedical Engineering, Southern Illinois University Carbondale, Carbondale, IL 62901, USA; n.talluri@siu.edu; 2 Department of Mechanical Engineering and Energy Processes, Southern Illinois University Carbondale, Carbondale, IL 62901, USA; k.amar@siu.edu (K.A.); tasnim.shireen@siu.edu (T.S.); 3 Carl R. Woese Institute for Genomic Biology, University of Illinois at Urbana-Champaign, Urbana, IL 61801, USA; Michael.Saul@jax.org; 4 Microbiology Program, Southern Illinois University Carbondale, Carbondale, IL 62901, USA; vjollca@micro.siu.edu; 5 Computational Biology, School of Computer Science, Carnegie Mellon University, Pittsburgh, PA 15213, USA; jianma@cs.cmu.edu; 6 Department of Biophysics and Biophysical Chemistry, Johns Hopkins University, Baltimore, MD 21205, USA; tjha@jhu.edu

**Keywords:** melanoma, 3D-fibrin gel, tumor repopulating cells, RNA-sequencing, biomarkers

## Abstract

Soft 3D-fibrin-gel selected tumor repopulating cells (TRCs) from the B16F1 melanoma cell line exhibit extraordinary self-renewal and tumor-regeneration capabilities. However, their biomarkers and gene regulatory features remain largely unknown. Here, we utilized the next-generation sequencing-based RNA sequencing (RNA-seq) technique to discover novel biomarkers and active gene regulatory features of TRCs. Systems biology analysis of RNA-seq data identified differentially expressed gene clusters, including the cell adhesion cluster, which subsequently identified highly specific and novel biomarkers, such as *Col2a1*, *Ncam1*, *F11r*, and *Negr1*. We validated the expression of these genes by real-time qPCR. The expression level of *Col2a1* was found to be relatively low in TRCs but twenty-fold higher compared to the parental control cell line, thus making the biomarker very specific for TRCs. We validated the COL2A1 protein by immunofluorescence microscopy, showing a higher expression of COL2A1 in TRCs compared to parental control cells. KEGG pathway analysis showed the JAK/STAT, hypoxia, and Akt signaling pathways to be active in TRCs. Besides, the aerobic glycolysis pathway was found to be very active, indicating a typical Warburg Effect on highly tumorigenic cells. Together, our study revealed highly specific biomarkers and active cell signaling pathways of melanoma TRCs that can potentially target and neutralize TRCs.

## 1. Introduction

Cancer metastasis is the leading cause of cancer-related deaths [1,2,3]. During metastasis, cells from the primary tumor spread to distant secondary sites in the body. Within the primary confinement of the tumor, there exists a heterogeneous tumor cell population [4,5,6,7,8]. However, not all cells within the primary tumor can sustain the journey to successfully repopulate in distant secondary sites. It is widely believed that “stem-cell-like” cancer cells or highly tumorigenic cells within the tumor possess unlimited self-renewal capacity and replicative immortality [9,10,11]. These self-renewing cancer cells, present in low numbers within the tumor, have the capacity to survive and repopulate secondary sites.

Although identifying these self-renewing cancer cells is an area of great interest to cancer biology, the conventional biomarker-based approach still remains a bottleneck problem due to unreliable surface antigen markers [12,13]. A recent report demonstrated the feasibility of selecting such highly tumorigenic subpopulation by a label-free method [14]. Single mouse melanoma cells were cultured in soft 3D fibrin gels with stiffness ~90 Pa [14] that mimic soft physiological tissues [15]. Within a few days of culture in soft 3D fibrin gel, 8–10% of the planted cells established multicellular spheroids. These cells within the spheroids were found to be highly tumorigenic and, thus, were aptly named as tumor repopulating cells (TRCs) [14,16,17,18]. However, very little is known about the specific and reliable biomarkers of TRCs and their molecular and genetic features. By using a next-generation sequencing (NGS)-based RNA-sequencing (RNA-seq) method, we pursued exciting research at the scientific interface between cell mechanics and genomics technology to identify TRC-specific novel biomarkers and their genetic features.

## 2. Experimental Section

### 2.1. 3D Fibrin Gel Fabrication

Salmon Fibrinogen and thrombin (Reagent Proteins) were used to setup the 3D-fibrin-gel cell culture. The TRC isolation procedure with 3D fibrin gel has been described elsewhere [14]. Briefly, fibrinogen was diluted to 2 mg/mL with the T7 buffer consisting of 50 mM Tris and 150 mM NaCl with pH adjusted to 7.4. Fibrinogen and DMEM complete cell culture medium were mixed at a ratio of 1:1 to finally obtain 1 mg/mL fibrinogen concentration (for 90 Pa fibrin gels). In total, 1000 cells were seeded in each of the 96 wells, which were pre-incubated with 100 U/mL thrombin. The 96-well tissue culture plate was placed into a 37 °C cell culture incubator for 15–20 min to ensure the polymerization of the fibrin gel. Finally, 200 µL of cell culture medium was added into each well. Fresh culture medium was exchanged every two days. TRCs were freshly isolated from 3D fibrin gels on day 5.

### 2.2. RNA-seq Data Collection

RNA-seq data on TRCs and parental B16F1 control cell lines were collected by the High-Throughput Sequencing and Genotyping Unit in Roy J. Carver Center for Biotechnology at the University of Illinois at Urbana-Champaign. Briefly, RNA samples were quality controlled for purity, integrity, and concentration. All RNA samples used for sequencing had RNA integrity numbers greater than 9.5. Libraries were made using an Illumina TruSeq Stranded RNA Sample Prep kit according to the manufacturer’s protocols, using four distinguishable adapter barcodes. The four libraries were pooled in equimolar concentrations, then sequenced in single end format on two lanes of an Illumina HiSeq 2500 sequencer using a TruSeq SBS sequencing kit v1 for 101 cycles.

The resulting sequence files were converted into FASTQ format and demultiplexed using CASAVA v1.8.2. Read depth ranged from 50,029,350 to 61,372,111 reads. Sequence data files were deposited in the Gene Expression Omnibus and can be accessed at the following accession number: GSE130697.

### 2.3. RNA-seq Bioinformatics and Differential Expression Analysis

The resulting demultiplexed FASTQ files for the RNA-seq dataset were analyzed using standard tools. Raw FASTQ files were trimmed using Trimmomatic v0.35 running on Java v1.8.0_72 in single end mode. Trimmed files were aligned with the GRCm38 mouse genome with Ensembl v75 annotation. Alignment was performed with Tophat2 v2.1.0, which utilized Bowtie2 v2.2.6 and Samtools v1.3. Reads were counted using htseq-count from the HTSeq Python framework v0.6.0 in Python v2.7.10.

Differential expression analysis was performed with the edgeR Bioconductor package v3.12.0 [19] in R v3.2.0. Briefly, tag counts were imported, meta-tags and low-expressed tags (less than 1 CPM expression in at least 3 samples) were filtered out, and samples were TMM normalized. TRC data were compared to control cells using the exactTest() function with tagwise dispersion estimates, and the final top table containing 11,208 genes was corrected for false discovery rate (FDR) using the Benjamini–Hochberg method [20] and FDR threshold <0.05. Results were annotated for gene information using the Ensembl v75 database accessed by the biomaRt Bioconductor package v2.26.1.

All bioinformatics and data analysis steps were performed on OS X v10.11.2.

### 2.4. DAVID, Gene Ontology, and KEGG Pathway Analyses

To identify higher order biological systems implicated in TRCs, we used DAVID, Gene Ontology (GO), and KEGG pathway enrichment analysis. We used DAVID v6.8 [21] to initially identify the clusters gene systems differentially expressed in TRCs. We then used the up- and downregulated differentially expressed genes in the topGO Bioconductor package v2.22.0 with the weight algorithm and the Fisher test statistic. GO Biological Process visualization utilized GO semantic similarity to visualize semantically similar clusters. Briefly, distances were calculated using the Rel similarity method with the GOSemSim Bioconductor package v1.28.2 [22]. Up- and downregulated GO clusters were visualized using the isoMDS function in MASS v7.3-45. KEGG pathway enrichment analysis utilized the gage Bioconductor package v2.20.1 [23] on log_2_-transformed data. To visualize the data, we used the pathview Bioconductor package v1.10.1 [24].

## 3. Results

### 3.1. Growth and Selection of TRC Spheroids by 90 Pa Stiffness 3D Fibrin Gel

**Figure 1:**
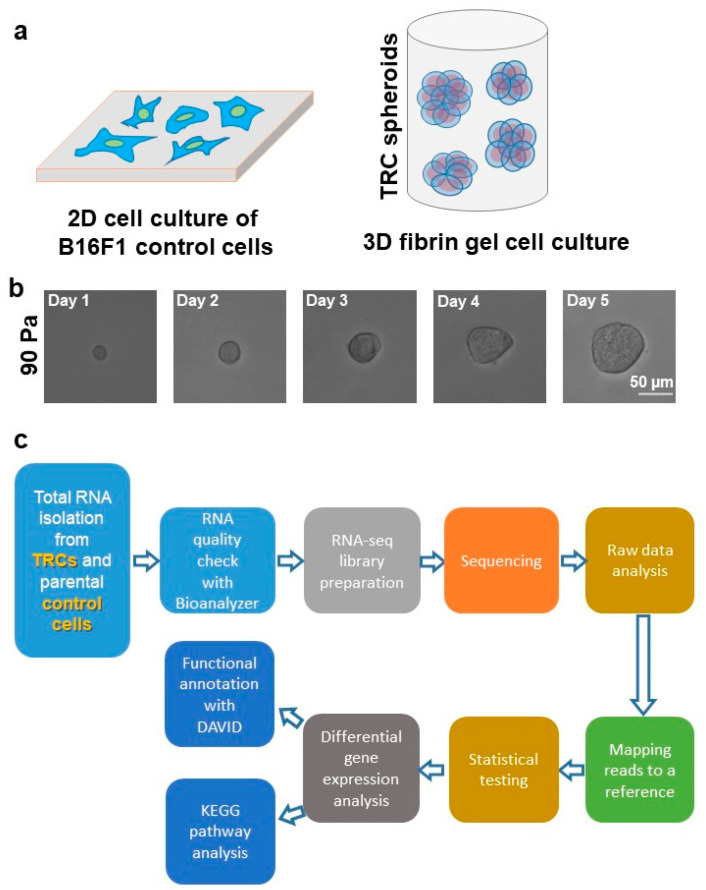
Isolation and differential gene expression analysis of soft 3D fibrin gel selected TRCs. (**a**) A schematic displaying 2D culture condition of parental B16F1 control cells and TRC spheroid generation in soft 3D fibrin gel. (**b**) Single B16F1 parental control cells, when cultured in 3D fibrin gel of 90 Pa stiffness, grow into multicellular spheroids. A tracked single cell is shown here giving rise to a TRC spheroid in 5 days. (**c**) A pipeline of RNA-seq data analysis is shown here.

The cell–cell and cell–matrix interactions are different in 2D vs. 3D culture conditions because of the spatial arrangements (Figure 1a). Furthermore, critical cellular functions, such as cell growth, proliferation, spreading, crawling, migration, and contractility, are regulated by not only in 2D and 3D microenvironments but also by the physical properties of the microenvironment, such as stiffness [25]. Established cell lines, such as parental B16F1 cells, are traditionally cultured on tissue 2D culture treated rigid dishes. Previous studies, including our own, demonstrated that 3D fibrin gel of 90 Pa stiffness promotes the growth and selection of multicellular TRC spheroids [14,16,17,18]. Growing evidence also reports similar findings that physical properties of the cellular microenvironment, such as stiffness, have an important role in promoting tumorigenic cancer cell growth [26,27]. Based on this, we seeded single B16F1 melanoma cells in 90 Pa soft 3D fibrin gels. The majority of the cells do not survive the soft 3D microenvironment, which is consistent with published reports [14,16,17,18]. We tracked and imaged single cells up to 5 days (Figure 1b). Figure 1b shows a single tracked cell growing into a multicellular TRC spheroid over a 5-day culture period. These mechanically selected TRCs have been shown to be highly tumorigenic [14,16,17,18]. However, it remains largely unknown how these tumorigenic TRCs are different from parental B16F1 control cells in terms of gene expression and gene regulatory network. To obtain a thorough understanding of differentially expressed genes of TRCs compared to parental B16F1 control cells, we profiled the whole transcriptome by the next-generation sequencing based RNA sequencing (RNA-seq) technique. Figure 1c shows the flow diagram of RNA-seq and subsequent data analysis strategy. Total RNA was isolated from parental B16F1 cells and soft 3D fibrin derived TRCs. The total RNA purity and integrity were evaluated by RNA integrity number (RIN) and subsequently, the RNA-seq library was prepared for sequencing. The raw RNA-seq data were mapped to a reference sequence and differentially expressed genes (DEGs) were obtained after statistical testing. Finally, for all DEGs, GO analysis and pathway enrichment analysis were carried out by the online tools DAVID and KEGG pathway analysis, respectively.

### 3.2. Identification of DEGs between Parental B16F1 Cells and 3D Fibrin-Derived TRC Spheroids

**Figure 2:**
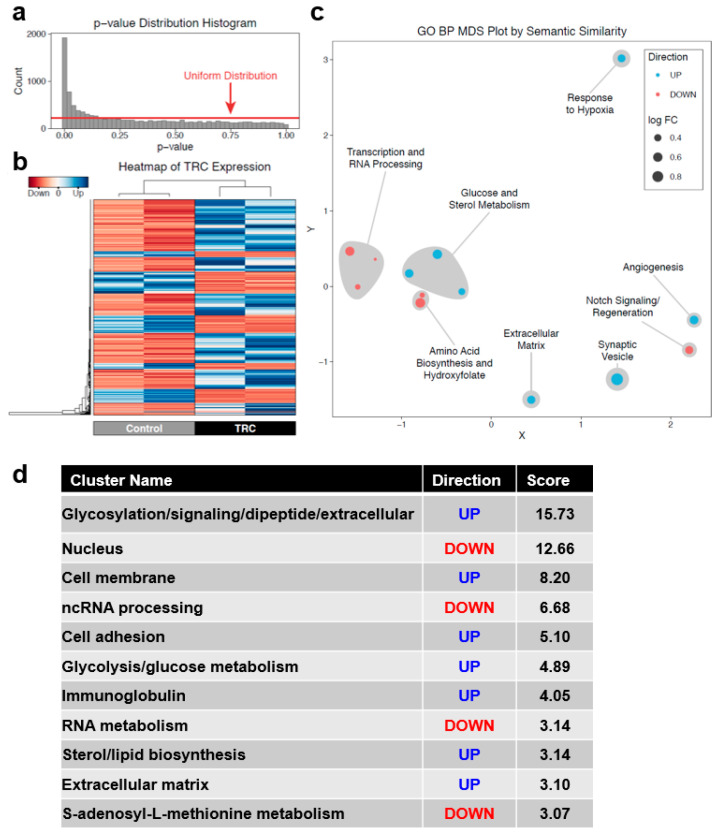
A summary of the RNA-seq data is reported here. (**a**) The RNA-seq experiment demonstrates significant deviation away from the theoretical uniform distribution (red line) with a spike towards zero, indicating that there are marked alterations in global expression measured in this experiment. (**b**) Genes differentially expressed at FDR < 0.05 and expression ≥ two-fold show strong differences between TRCs and parental control cells. (**c**) Non-metric MDS on semantic similarity of GO Biological Process terms altered in TRCs relative to parental control cells demonstrates a few discrete clusters of terms related to assorted tumor processes. (**d**) A summary of upregulation (blue) and downregulation (red) of different clusters is shown here.

The differential expression results yielded a *p*-value distribution with deviation away from the uniform distribution expected under the null hypothesis and a spike at 0, indicating that the transcriptional state of TRCs differs strongly from parental B16F1 control samples (Figure 2a). A comparison of TRC transcriptome to parental B16F1 control identified a total of 1809 differentially expressed genes at FDR < 0.05. Of these genes, 1004 were upregulated and 805 were downregulated. Imposing an additional filter of two-fold or greater change identified 649 upregulated and 296 downregulated genes (Figure 2b). Figure 2b shows a heatmap of the gene expression profile of TRCs and parental B16F1 control cells. Overall, these results demonstrate that a large disparity in gene expression exists between TRCs and parental control cells.

Gene Ontology enrichment proceeded with genes differentially expressed without a fold-change filter to fully identify differentially modulated systems. This analysis identified upregulated Biological Process clusters in TRCs related to glucose and sterol metabolism, hypoxia, synaptic vesicles, angiogenesis, and extracellular matrix (Figure 2c). These Biological Process clusters indicate that TRCs show signs of Warburg Effect, a shift seen in very tumorigenic cells wherein metabolism shifts away from oxidative phosphorylation and towards aerobic glycolysis [28,29].

Downregulated Biological Process clusters included transcription and RNA processing, amino acid biosynthesis, and Notch signaling-based regeneration. The downregulation of transcription, RNA processing, and amino acid biosynthesis suggests that these cells prioritize metabolic activities related to repopulation, relative to other cell populations. Since TRCs are assumed to undergo regeneration, the downregulated regeneration GO term appears to be counterintuitive. After looking into the set of genes belonging to the regeneration term, we find that the genes belong to the Notch signaling pathway, which are found to be downregulated in TRCs, and are consistent with a previous study [30]. Therefore, we posit that TRCs utilize separate pathways for regeneration. In addition, we used DAVID [21] to find clusters of enriched functional terms. Figure 2d shows a summary of different clusters with upregulation and downregulation. These systems’ biology analyses discovered multiple interesting gene regulatory features and candidate biomarkers of TRCs.

### 3.3. COL2A1 Is a Highly Specific Biomarker of Melanoma TRCs

The cell adhesion cluster revealed top candidates that can serve as novel and specific biomarkers of TRCs, as shown in Table 1. The top biomarkers include *Col2a1*, *Ncam1*, *F11r*, *Col11a2*, *Negr1*, and *Cd47*. Among these biomarkers, the expression level of *Col2a1* was found to be relatively low in TRCs but approximately twenty-fold higher compared to parental B16F1 control cells. This suggests that *Col2a1* is selectively expressed in TRCs.

**Figure 3:**
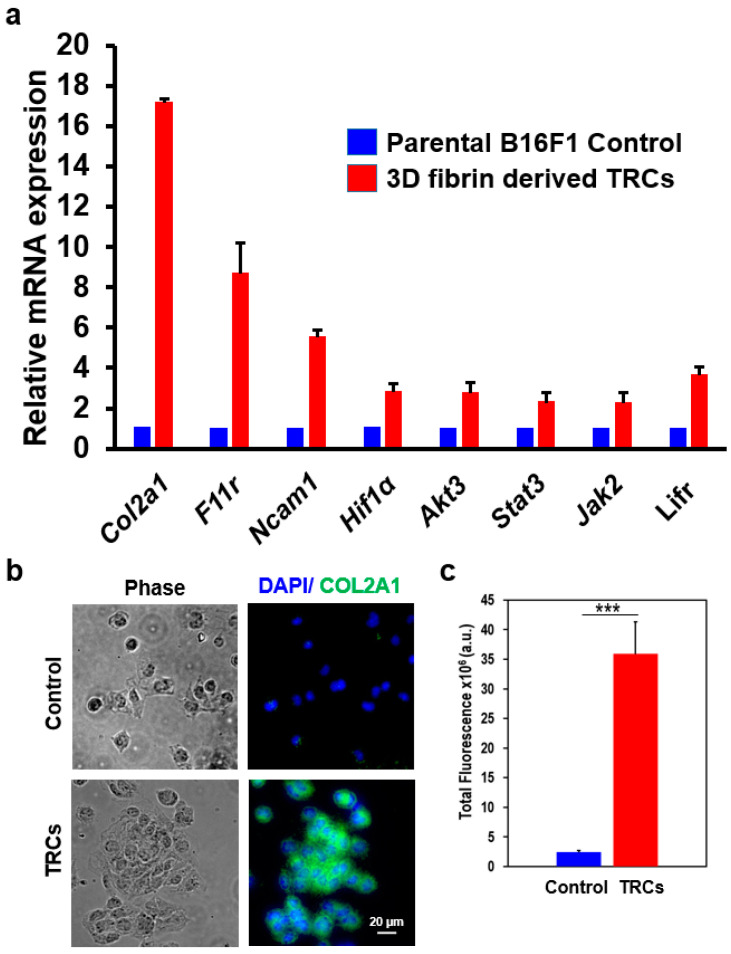
Validation of differential expressed genes of TRCs. (**a**) Quantification of candidate biomarkers and differentially expressed genes of TRCs by quantitative real-time PCR experiments. Data represent mean ± s.e.m. and from three independent experiments. A 2^−ΔΔ*C*t^ method was used for gene expression with each gene *Ct* normalized by the *Ef1a* housekeeping gene. No reverse transcriptase control was used as a negative control. (**b**) Validation of the COL2A1 protein expression by immunofluorescence microscopy. Cells were fixed and labeled with anti-COL2A1 primary antibody followed by FITC-conjugated secondary antibody and counterstained with DAPI. COL2A1 signal was much higher in fixed TRCs (2nd row) compared to fixed parental control cells (1st row). (**c**) The total fluorescence signal of anti-COL2A1: FITC of single TRCs was found to be significantly higher compared to parental control cells and presented in a bar blot (n = 25 for both TRC and control cells; *** represents *p* < 0.001).

To validate the RNA-seq results, next, we performed real-time qPCR on top 3 highly expressed cell adhesion related transcripts, namely *Col2a1*, *F11r*, and *Ncam1* as shown in Figure 3a. The PCR primer sequences are shown in the Supplementary Table S1. The results shown in Figure 3a are from three biological replicates. The expression level of all three genes tested shows a close correlation between qPCR and RNA-seq data. The qPCR data show that *Col2a1* expression level is seventeen-fold higher in TRCs when compared to parental B16F1 control cells. We further validated the COL2A1 protein expression by immunofluorescence microscopy. When TRCs and their parental B16F1 control cells were fixed and stained with anti-COL2A1 antibody, we found an approximately 14-fold higher total fluorescence signal in TRCs compared to parental control cells (Figure 3b-c). The expression level of *F11r* and *Ncam1* were found to be approximately nine-fold and six-fold higher in TRCs, respectively, which correlates well with the RNA-seq data. Furthermore, we validated *Col2a1* expression in a highly metastatic B16F10 cell line, a metastatic derivative of B16F1 cell line. The immunocytochemistry assay with anti-COL2A1 antibody in B16F10 cell line revealed that *Col2a1* expression in B16F10 TRCs is indeed much higher than parental control cells, consistent with our B16F1 cell line findings (Supplementary Figure S1). However, we observed slightly higher expression of *Col2a1* in B16F10 parental control cells compared to B16F1 parental control cells. This might be due to the presence of more highly metastatic cells within the B16F10 cell population.

### 3.4. A Unique Set of Gene Regulatory Features Exists in TRCs

Table 1 lists cell signaling pathways that may regulate TRC self-renewal, growth, and survival. *Trp53* was among the 34 genes found in a cluster of genes generally associated with nuclear localization and transcriptional regulation. *Trp53* was found to be downregulated in TRCs, although previous research indicated that parental B16F1 melanoma cell line maintains a functional level of TRP53 [31,32]. The JAK/ STAT pathway is generally associated with the self-renewal of stem cells [33,34,35,36]. Interestingly, members of the JAK/ STAT pathway, namely, *Jak2*, *Stat3*, and *Lifr* were found to be upregulated in TRCs as shown by the RNA-seq data (Table 1). When we performed the real-time qPCR to validate the expression level of *Jak2*, *Stat3*, and *Lifr*, we found a good agreement with the RNA-seq data (Table 1 and Figure 3a). Both *Jak2* and *Stat3* were found to be upregulated by more than two-fold and *Lifr* by approximately four-fold (Figure 3a). In addition, RNA-seq data also suggest the upregulation of a gene related to the PI3K/ Akt pathway—specifically *Akt3*—which may, in part, contribute to TRC growth and proliferation. The real-time qPCR results also showed that *Akt3* was upregulated in TRCs by approximately three-fold. RNA-seq data do not suggest any change in the expression level of other members of the Akt pathway, such as *Akt1/2* (Table 1). Finally, hypoxia and angiogenesis-related genes, such as *Hif1a* and *Vegfa,* respectively, were found to be upregulated in TRCs but not in parental B16F1 control cells (Table 1 and Figure 3).

## 4. Discussion

The identification of dependable biomarkers is critical for the specific targeting of tumorigenic cancer cells. Soft 3D fibrin gel selected TRCs, a subpopulation of the B16F1 cell line, have been demonstrated to be highly tumorigenic. The expression level of *Cd133*, a cancer stem cell marker, in TRCs was found to be identical to parental B16F1 control cells [14]. Therefore, conventional biomarkers, such as CD133, cannot be used to reliably select and target TRCs. Our current study reveals a highly specific and novel suite of biomarkers of TRCs including the top candidate *Col2a1*. Real-time qPCR data and immunocytochemistry data validated that the *Col2a1* transcript and the protein it encodes are indeed specifically expressed in TRCs. It is interesting to note that *Col2a1* encodes a component of type-II collagen, named the pro-alpha1(II) chain. It is first expressed during eye development and in the cartilage cells of a fully developed animal. Besides the adhesion supporting role, *Col2a1* has been associated with cell signaling pathways, such as transforming growth factor-β (TGF-β)/Smad signaling, which is known to promote metastasis [37,38,39]. Moreover, similar to the cartilage matrix, which is rich in type II collagen, the building blocks of the tumor niches may be provided by the availability of type II collagen component, such as COL2A1. Therefore, it is not unreasonable to implicate the tumor repopulating cells’ ability to engineer the tumor niche. A recent report also demonstrates a similar upregulation in *Col2a1* expression in breast cancer cells [40].

The RNA-seq data revealed the cryptic and unique gene regulatory features of TRCs. *Trp53* is a transcriptional regulator involved in cell growth and apoptosis and has been well characterized as a tumor suppressor. Although it is well documented that parental B16F1 cells have a functional *Trp53* gene [31,32], our RNA-seq data found an approximately two-fold downregulation of *Trp53* expression in TRCs, potentially revealing a mechanism underlying TRC tumorigenicity. Consistent with our findings, a recent study suggests that the expression level of *Trp53* in TRCs was lower than parental B16F1 cells [41]. PI3K/ Akt pathway is involved in cell growth, proliferation, and survival among many other critical functions. Our study shows similar *Akt1/2* kinase expression level in both parental control cells and TRCs. Consistent with our results, a previous study demonstrated that inactivation of *Akt1/2* kinase had very little effect on TRC growth and proliferation [14]. When we further investigated the possible upregulation of other Akt kinases that could be linked to the proliferation of cancers, including melanoma [42,43,44], we found *Akt3* to be upregulated in TRCs by more than two-fold (Table 1 and Figure 3a). In metastatic melanomas, it has been reported in the past that the selective activation of *Akt3*, but not *Akt1/2*, increases the survival rate of tumorigenic cells and tumor development in 40–60% of non-familial melanomas [45]. It is interesting to note that the sterol biosynthesis pathway is very active in TRCs (Supplementary Figure S2). Currently, we do not know why TRCs behave like this and we will investigate this in the future.

Generally, mouse pluripotent stem cells rely on self-renewal via the JAK/ STAT pathway [34,36]. Our RNA-seq data show TRCs expressing upregulated genes related to the JAK/STAT pathway (Table 1, Figure 3, and Supplementary Figure S3). Consistent with our findings, past studies have indicated that many types of cancers, including melanoma, involve the active JAK/STAT pathway [46,47]. This may be due to the acquisition of cancer-stem-cell-like self-renewal properties by the active JAK/STAT pathway. The translocation of STAT3 protein allows direct binding to DNA promoting cell growth, survival, anti-apoptosis, migration and metastasis [48,49,50]. Furthermore, a recent study suggests that STAT3 activation also perturbs the dormancy of melanoma tumor repopulating cells [51]. Our RNA-seq data identified an approximately two-fold upregulation of both *Jak2* and *Stat3* in TRCs. However, it is beyond the scope of this work to undertake detailed analysis and perturbation of JAK/STAT pathway as a therapeutic approach for targeting melanoma TRCs.

TRCs display a unique growth advantage over non-TRCs. As with many metastatic cancers, TRCs show an upregulation of glycolysis pathway, as is evident from our KEGG pathway data analysis (Supplementary Figure S4). The genes associated with the glycolysis pathway are observed to be highly upregulated. This phenomenon, also referred to as the Warburg Effect, is a known hallmark of many cancers. Cells exhibiting the Warburg Effect produce energy via glycolysis followed by lactic acid fermentation, regardless of the availability of oxygen. Our data also indicate TRC adaptation to even a low oxygen environment, possibly via the upregulated HIF1A/hypoxia pathway. Together, we propose that a higher rate of metabolism of glucose leading to lactic acid production even in hypoxic conditions provides TRCs a growth advantage over non-TRCs. It has been speculated that the upregulation of glycolysis leading to lactic acidosis allows for the evolution of a cell subpopulation that becomes resistant to even acid-induced cell toxicity. Our soft 3D-fibrin-gel selected TRCs match this description fairly well. Our data indicates that TRCs with upregulated glycolysis and acidic microenvironment resistance display a strong growth advantage, thus promoting proliferation and tumorigenicity.

## 5. Conclusions

In conclusion, our study revealed a set of a highly specific and novel biomarkers of melanoma TRCs in addition to active signaling pathways related to B16F1 melanoma TRC self-renewal, growth, proliferation, and survival. For the neutralization of TRCs, the perturbation of signaling pathways, such as JAK/STAT and PI3K/Akt3, will be studied in the future more comprehensively. Moreover, the presence of the COL2A1 protein in human melanoma tumor sections will be validated in the future. Future studies will be designed to unveil the role of biomarkers, such as COL2A1, F11R, NCAM1, and CD47, for the successful targeting and neutralization of TRCs.

## Figures and Tables

**Table 1:** TRC candidate biomarkers and differentially expressed genes related to various signaling pathways.

	Gene Name	Fold Change
**Cell surface biomarkers**	*Col2a1*	20.82
	*Ncam1*	7.7
	*F11r*	4.41
	*Col11a2*	3.45
	*Negr1*	2.64
	*Cd47*	2.31
**p53 pathway**	*Trp53*	−2.07
**JAK/ STAT pathway**	*Lifr*	4.47
	*Jak2*	1.51
	*Stat3*	1.97
**Hypoxia related genes**	*Hif1a*	1.96
**Angiogenesis**	*Vegfa*	4.36
**PI3K/ Akt pathway**	*Akt1*	1.04
	*Akt2*	1.23
	*Akt3*	2.25

## Supplementary Materials

The following are available online at https://www.mdpi.com/2227-9059/8/9/360/s1, Supplementary Experimental Procedures, Table S1: Forward and reverse primer sequences, Figure S1: Upregulation of COL2A1 in B16F10 TRCs, Figure S2: Upregulation of steroid synthesis in TRCs, Figure S3: The chemokine signaling pathways were found to be active in TRCs, and Figure S4: The glycolysis pathway is very active in TRCs.
