# Supplementary material for: COL2A1 Is a Novel Biomarker of Melanoma Tumor Repopulating Cells
Source: Biomedicines. 2020 Sep 18;8(9):360. doi: 10.3390/biomedicines8090360 (PMC7555262; doi:10.3390/biomedicines8090360)
Supplement: Supplementary file 1 [file biomedicines-08-00360-s001.pdf]

Supplementary Information for "COL2A1 is a Novel Biomarker of Melanoma Tumor Repopulating Cells"

Bhavana Talluri[1*], Kshitij Amar[2*], Michael Saul[3], Tasnim Shireen[2†], Vjollca Konjufca[4], Jian Ma[5], Taekjip Ha[6], Farhan Chowdhury[1,2‡]

[1]Biomedical Engineering Program, Southern Illinois University Carbondale, Carbondale, IL 62901, U.S.A.; [2]Department of Mechanical Engineering and Energy Processes, Southern Illinois University Carbondale, Carbondale, IL 62901, U.S.A.; [3]Carl Woese Institute for Genomic Biology, University of Illinois at Urbana-Champaign, Urbana, IL, 61801, U.S.A.; [4]Department of Microbiology, Southern Illinois University Carbondale, Carbondale, IL 62901, U.S.A.; [5]Computational Biology, School of Computer Science, Carnegie Mellon University, Pittsburgh, PA, 15213, U.S.A.; [6]Department of Biophysics and Biophysical Chemistry, Johns Hopkins University, Baltimore, MD 21205, U.S.A. [*]Contributed equally. [†]Present address: Department of Mechanical and Aerospace Engineering, State University of New York- Buffalo, Buffalo, NY, U.S.A.

[‡]Correspondence: farhan.chowdhury@siu.edu (F.C.)

This PDF file includes additional

- Experimental Procedures [Pg. 2-3]

- Supplementary Table [Pg. 4]

- Supplementary Figures [Pg. 5-8]

- References [Pg. 9]

SUPPLEMENTARY EXPERIMENTAL PROCEDURES

Total RNA isolation and purification from cells

Total RNA, from freshly isolated TRCs, was extracted and purified using Qiagen's RNeasy Mini Kit (Qiagen; cat. # 74104) according to manufacturer's protocol. Briefly, parental B16F1 control cells and soft 3D fibrin derived TRCs were harvested and spun down into cell pellets. The lysing buffer was added to the pellets and vortexed vigorously. Then, the lysates were passed through QiaShredder columns (Qiagen; cat. # 79654) and followed by spinning down at maximum speed for 2 minutes. The flow-through was collected and 70% ethanol was added before each sample ran through RNeasy mini columns. The flow-through was discarded and RNeasy columns were washed with RW1 and RPE buffers. Finally, RNase-free water was added to the column membrane to elute RNA. The purified RNA concentration was measured using a NanoDrop spectrophotometer.

Quantitative real-time PCR experiment

Total RNA was extracted as described in the previous step. During RNA handling steps, isolated and purified total RNA was kept on ice to inhibit RNA degradation. The mRNA was converted to cDNA using the BioRad's iScript cDNA synthesis kit (Bio-Rad Laboratories, Hercules, CA; cat # 1708841). The quantitative PCR was performed using SYBR Green PCR Master Mix (Bio-Rad Laboratories, Hercules, CA, cat # 172-5270). The expression level of genes, namely, Col2a1, F11R, NCAM1, HIF1α, Akt3, STAT3, JAK2, and LIFR was examined. For relative mRNA expression analysis, the cycle threshold (C_T) value for samples was determined. These C_T values were then normalized with respect to elongation factor alpha1 (Ef1α), as the housekeeping gene, for relative gene-expression quantification. The primer sequences of the genes tested are shown in supplementary Table S1.

Immunofluorescence microscopy

Parental B16F1 control cells and TRCs were fixed with 4% paraformaldehyde for 10 min at room temperature as described before [1]. Fixed cells were permeabilized with 0.1% Tween and blocked with 3% BSA in PBS for 1 hour at room temperature. Cells were incubated with primary antibody (Rabbit anti-col2a1, Abcam ab34712) at 1:200 dilution for 1.5 hours followed by incubation of goat anti-rabbit secondary antibody conjugated with FITC dye (EMD Millipore AP307F) at a dilution of 1:500 for 1 hour. Liquid antifade mounting medium (SlowFade™ Diamond Antifade Mountant, Thermo Fisher Scientific S36963) was directly applied to the fluorescently labeled cells before imaging.

SUPPLEMENTARY TABLES

Supplementary Table S1: Forward and reverse primer sequences

	Forward	Reverse
Col2a1	5'-GGG AAT GTC CTC TGC GAT GAC-3'	5'-GAA GGG GAT CTC GGG GTT G-3'
F11r	5'-TAC GAG AAC ACT GCC CCC TAC-3'	5'-TCC ACA ACC AAT GTC AGC GAC-3'
Ncam1	5'-GAC AGA ACC CGA AAA GGG C-3'	5'-GTT GGG GAC CGT CTT GAC TT-3'
Hif1a	5'-ACC TTC ATC GGA AAC TCC AAA G-3'	5'-ACT GTT AGG CTC AGG TGA ACT-3'
Akt3	5'-TGG GTT CAG AAG AGG GGA GAA-3'	5'-AGG GGA TAA GGT AAG TCC ACA TC-3'
Stat3	5'-CAA TAC CAT TGA CCT GCC GAT-3'	5'-GAG CGA CTC AAA CTG CCC T-3'
Jak2	5'-TTG TGG TAT TAC GCC TGT GTA TC-3'	5'-ATG CCT GGT TGA CTC GTC TAT-3'
Lifr	5'-TAC GTC GGC AGA CTC GAT ATT-3'	5'-TGG GCG TAT CTC TCT CTC CTT-3'
Ef1α	5'-CAA CAT CGT CGT AAT CGG ACA- 3'	5' - GTC TAA GAC CCA GGC GTA CTT - 3'

SUPPLEMENTARY FIGURES

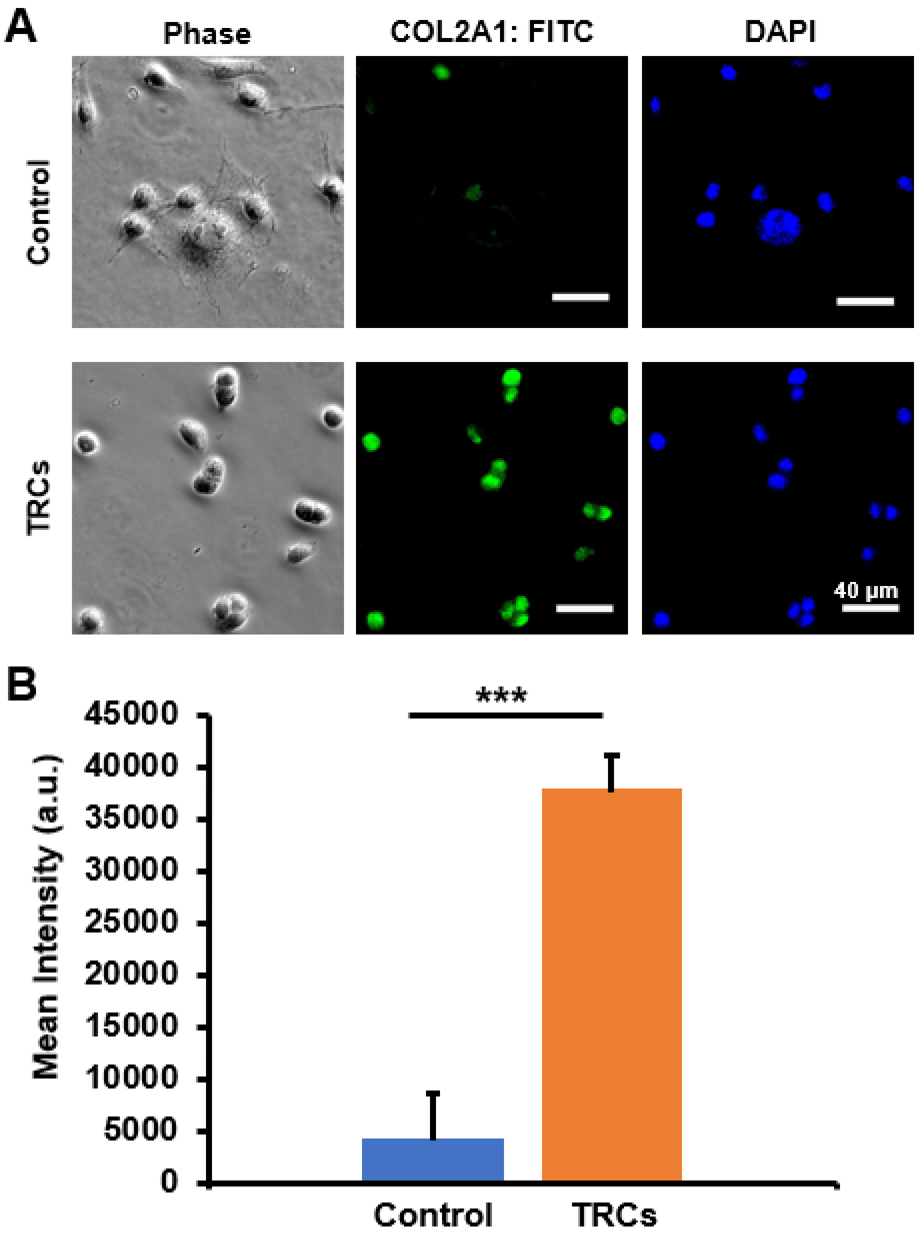

Supplementary Fig. S1. Upregulation of Col2a1 in B16F10 TRCs. (A) Single B16F10 cells were seeded in 90 Pa fibrin gel for five days. After 5 days, the B16F10 TRCs were freshly isolated and plated on rigid dishes. Cells were fixed with 4% paraformaldehyde and immunocytochemistry assay was performed with the anti-Col2a1 antibody. (B) The mean intensity quantification of B16F10 TRCs show an upregulation of Col2a1 fluorescence signal compared to control cells ($p < .001$). n= 14 for TRCs and n=15 for control cells.

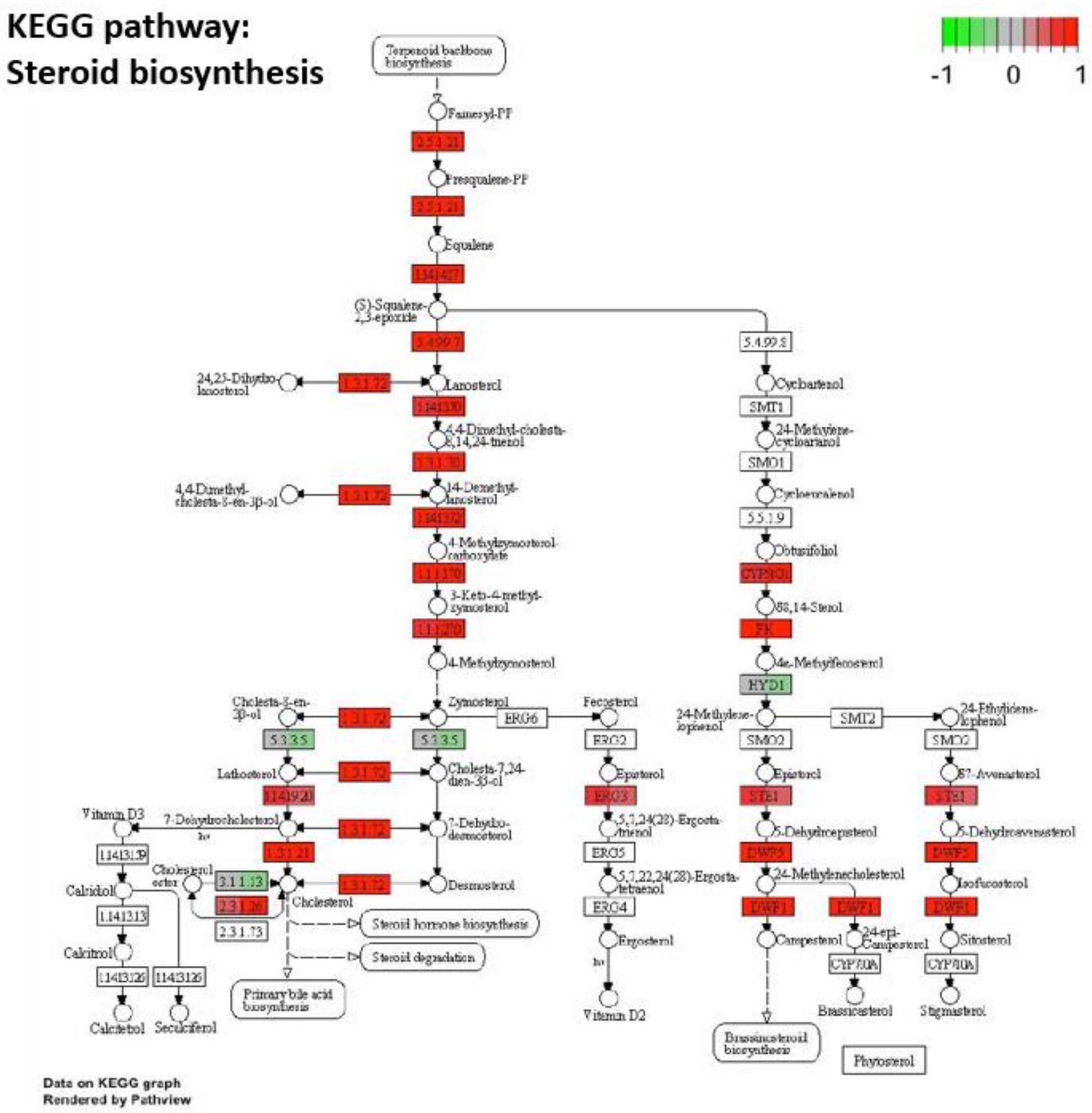

Supplementary Fig. S2. Upregulation of steroid synthesis in TRCs. KEGG pathway analysis of differentially expressed genes from RNA-seq data shows upregulation (red) of steroid synthesis in TRCs.

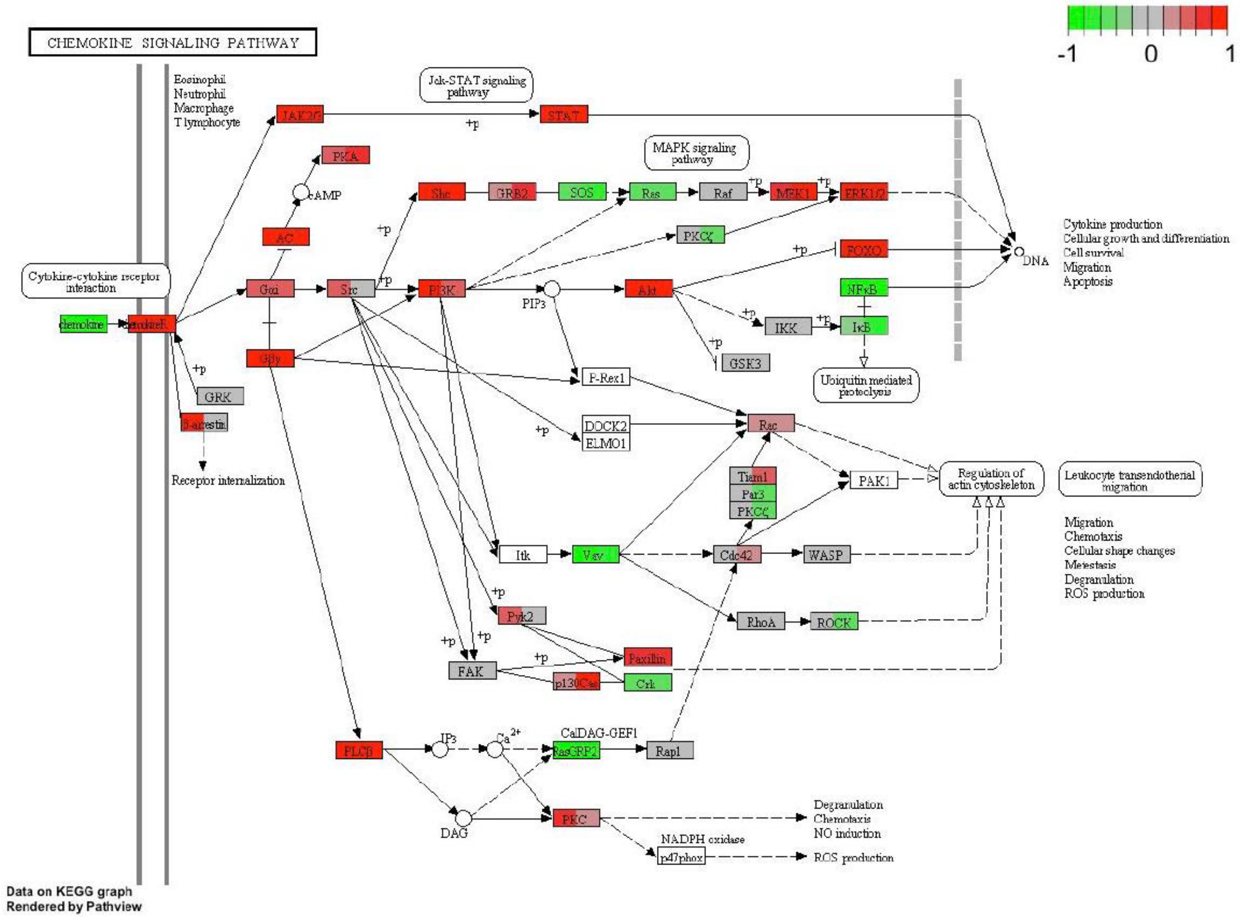

Supplementary Fig. S3. The chemokine signaling pathways were found to be active in TRCs. KEGG pathway analysis of RNA-seq data shows upregulation of genes (red) related to the JAK/ STAT pathway in TRCs.

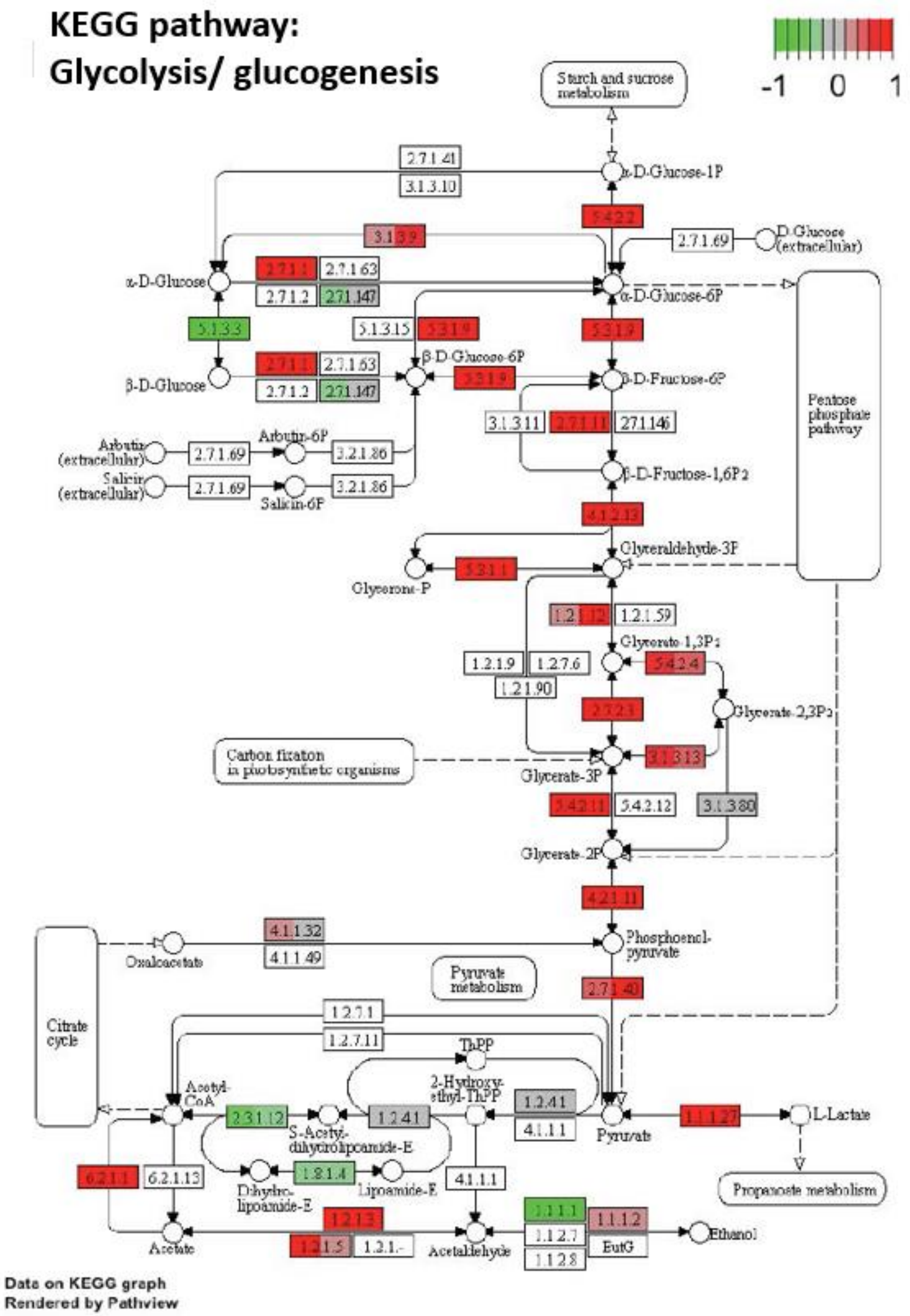

Supplementary Fig. S4. The glycolysis pathway is very active in TRCs. KEGG pathway analysis of RNA-seq data shows upregulation of genes (red) related to glycolysis/ glucogenesis in TRCs.

REFERENCES

1. Chowdhury, F.; Doğanay, S.; Leslie, B.J.; Singh, R.; Amar, K.; Talluri, B.; Park, S.; Wang, N.; Ha, T. Cdc42-dependent modulation of rigidity sensing and cell spreading in tumor repopulating cells. *Biochemical and Biophysical Research Communications* **2018**, *500*, 557-563, doi:10.1016/j.bbrc.2018.04.085.
